# Preparation of Ethyl Cellulose Microspheres for Sustained Release of Sodium Bicarbonate

**DOI:** 10.22037/ijpr.2019.1100651

**Published:** 2019

**Authors:** Jia-Hui Wu, Xiao-Juan Wang, Shu-Juan Li, Xiao-Ying Ying, Jing-Bo Hu, Xiao-Ling Xu, Xu-Qi Kang, Jian You, Yong-Zhong Du

**Affiliations:** *Institute of Pharmaceutics, College of Pharmaceutical Sciences, Zhejiang University, 866 Yuhangtang Road, Hangzhou, 310058, PR China.*

**Keywords:** Microspheres, Sodium bicarbonate, Stabilizer, Encapsulation, Sustained release

## Abstract

Sustained release of thermal-instable and water-soluble drugs with low molecule weight is a challenge. In this study, sodium bicarbonate was encapsulated in ethyl cellulose microspheres by a novel solid-in-oil-in-oil (S/O/O) emulsification method using acetonitrile/soybean oil as new solvent pairs. Properties of the microspheres such as size, recovery rate, morphology, drug content, and drug release behavior were evaluated to investigate the suitable preparation techniques. In the case of that the ratio of the internal and external oil phase was 1: 9, Tween 80 as a stabilizer resulted in the highest drug content (2.68%) and a good spherical shape of microspheres. After the ratio increased to 1: 4, the microspheres using Tween 80 as the stabilizer also had high drug content (1.96%) and exhibited a sustained release behavior, with 70% of drug released within 12 h and a sustained release of more than 40 h. Otherwise, different emulsification temperatures at which acetonitrile was evaporated could influence the drug release behaviour of microspheres obtained. This novel method is a potential and effective method to achieve the encapsulation and the sustained release of thermal-instable and water-soluble drugs with low molecule weight.

## Introduction

Different drugs have different physicochemical properties, so different formulations are chosen according to the drugs. Sustained release of thermal-instable and water-soluble drugs with low molecule weight is always a popular topic in pharmacy (1-3). Microspheres have been generally used to envelop water-soluble drugs for sustained release because drugs are dispersed in the skeleton of water-insoluble materials (4, 5). And various microsphere fabrication methods may influence the stability and release kinetics of microspheres (6, 7). Spray-drying as a method to fabricate microspheres is feasible and economical and it is widely used in pharmaceutical production (8-10). But during the preparation, the solvent was volatilized at a high temperature, so it’s not suitable for those thermal-instable drugs because drugs may be decomposed. In order to avoid high temperatures, the solvent evaporation process is widely used because the process can be carried out at a moderate temperature (11, 12). Generally, emulsions based on oil-in-water (O/W) emulsification are formed and then the internal oil phase is evaporated to precipitate the polymer material to form microspheres, as has been reported before (13-18). However, because the drug diffuses into the external aqueous phase, this method may cause low drug loading efficiency, especially for those drugs with low molecule weight and good water-solubility. And the obtained microspheres may have a fast drug release. It is exciting to note that the external aqueous phase can be replaced by organic solvents to form oil-in-oil (O/O) emulsion in the solvent evaporation process, which may bring better encapsulation efficiency and improve the drug release behavior due to the poor solubility of drugs in the external oil phase (19). A water-in-oil-in-oil (W/O/O) double emulsion solvent diffusion method is also capable to improve loading efficiency of a water-soluble drug and modulate release profile (20). On this basis, the drug can be mixed into the internal oil phase to form small particles, following by the preparation of an S/O/O emulsion (21, 22). In this method, drug is encapsulated in the form of particles, which may lead to a better drug release behavior. Therefore, this method is more suitable for the encapsulation of thermal-instable and water-soluble drugs with low molecule weight.

In the preparation of microspheres, ethyl cellulose has been used as a matrix material to achieve sustained release of drugs due to its chemical stability, water-insolubility, flexibility, and low price (23-25). Sodium bicarbonate was capable of neutralizing the lactic acid in tumor tissues, thus improving anti-tumor effect, because lactic acid plays an important role in resistance to glucose deprivation-induced death of cancer cells (26, 27). However, sodium bicarbonate has great water-solubility (9.7 g/100 mL in 20 °C) and can hardly be soluble in organic solvents. Besides, it is easily decomposed when heating. Therefore, sodium bicarbonate can be seen as a model thermal-instable and water-soluble drug with low molecule weight.

In the present study, a novel solid-in-oil-in-oil (S/O/O) emulsification method was used to fabricate microspheres employing ethyl cellulose (EC) as a water-insoluble polymer and sodium bicarbonate as a model drug, respectively. Considering properties of sodium bicarbonate, this method was used to improve encapsulation efficiency and achieve the sustained release of sodium bicarbonate. The purpose of this study was to prepare sodium bicarbonate-loaded EC microspheres and evaluate the effects of different preparation techniques such as stabilizer, ratio of internal, and external oil phase and emulsification temperature on physical and chemical properties of the microspheres such as size, recovery rate, morphology, drug content, and drug release behavior. Besides, this study provided a novel idea to achieve the encapsulation and the sustained release of thermal-instable and water-soluble drugs with low molecule weight.

## Experimental

*Materials*

Ethyl cellulose (EC), soybean oil, Tween 20, Tween 60, Tween 80, PEG 400, Pluronic F-68, and Span 80 were purchased from Aladdin Bio-chem Reagent Company. Acetonitrile, sodium bicarbonate and n-hexane were purchased from Sinopharm Chemical Reagent CO., Ltd. All the reagents employed in this study were analytical grade.

*Preparation of nanosuspensions of sodium bicarbonate*

Prescription of nanosuspensions was shown in Table 1. Firstly, EC and stabilizer were dissolved in acetonitrile. Then, sodium bicarbonate solution (80 g/L) was injected into acetonitrile under a rapidly stirring to form the nanosuspensions of sodium bicarbonate. The nanosuspensions were further subjected to sonication.

*Characterization of nanosuspensions*

*Particle size*

The particle size of nanosuspensions of sodium bicarbonate was analyzed by dynamic light scattering (DLS). Number average particle size and size distribution were obtained.

*Particle morphology*

Nanosuspensions of sodium bicarbonate were centrifuged and the supernatant was removed to obtain particles. These sodium bicarbonate particles were then washed by acetonitrile for three times. Particle morphology of sodium bicarbonate was determined by scanning electronic microscope (SEM, Hitachi SU-8010). These particles were coated with gold in a vacuum for three times before observation.

*Preparation of* *sodium bicarbonate-loaded EC microspheres*

Solvent evaporation process was used to form microspheres as mentioned above. The prescription of microspheres was shown in Table 2. 

The above nanosuspensions were added to a rapidly stirring soybean oil containing Span 80 to form an S/O/O emulsion which was continuously stirred to volatilize acetonitrile. Then, microspheres were obtained after centrifugation from soybean oil solution, fully washed with n-hexane, and naturally dried. Furthermore, several influencing factors in the process of microspheres formation were investigated, such as stabilizer, temperature, and ratio of internal oil phase and external oil phase.

*Characterization of the microspheres*

*Recovery rate*

Recovery rate was calculated through the following formula:

Recovery rate = M_MC_/M_material_

Where M_material_ is the total mass of ethyl cellulose and sodium bicarbonate used in the preparation of microspheres, while M_MC_ is the mass of microspheres collected after drying.

*Surface morphology and number average particle size*

Surface morphology of sodium bicarbonate-loaded EC microspheres was determined by a scanning electronic microscope (SEM, KYKY-EM3200) at high magnification. These microspheres were coated with gold in a vacuum for three times before being scanned by SEM. Number average particle sizes of microspheres were determinated through SEM images.

*Drug content*

The microspheres (20 mg) were dispersed in dichloromethane (2 mL) to dissolve ethyl cellulose. Then, deionized water (1 mL) and sulfuric acid solution (0.5 mL, 0.01 M) was added, followed by sonication for 2 min. Subsequently, the dispersion was boiled for 2 min to eliminate dichloromethane and carbon dioxide. After that, the aqueous solution was separated through filtering and the precipitation was washed with deionized water. The aqueous solution and wash solution were collected. The drug content was determinated by titration using sodium hydroxide titration solution after addition of phenolphthalein.

*In-vitro drug release*

Sodium bicarbonate-loaded EC microspheres suspended in deionized water (4 mL) were added to tubes and then placed in a shaking incubator (SHY-2A, Guoyu Co. Ltd., Changzhou, China) at 37 °C and 100 rpm. At predeterminated times, 1 mL of the solutions were collected and an equal volume of the deionized water was added back to keep a constant volume. 

The amount of sodium bicarbonate released from microspheres was determined by titration as described above.

## Results and Discussion

*Preparation and Characterization of sodium bicarbonate nanosuspensions*

In the preparation of microspheres, if the drug is encapsulated in the form of molecules, the resulting microspheres may exhibit a fast drug release behavior, as has been mentioned above. Therefore, we considered that sodium bicarbonate can be encapsulated into microspheres in the form of nanoparticles, in order to achieve better sustained release.

**Figure 1:**
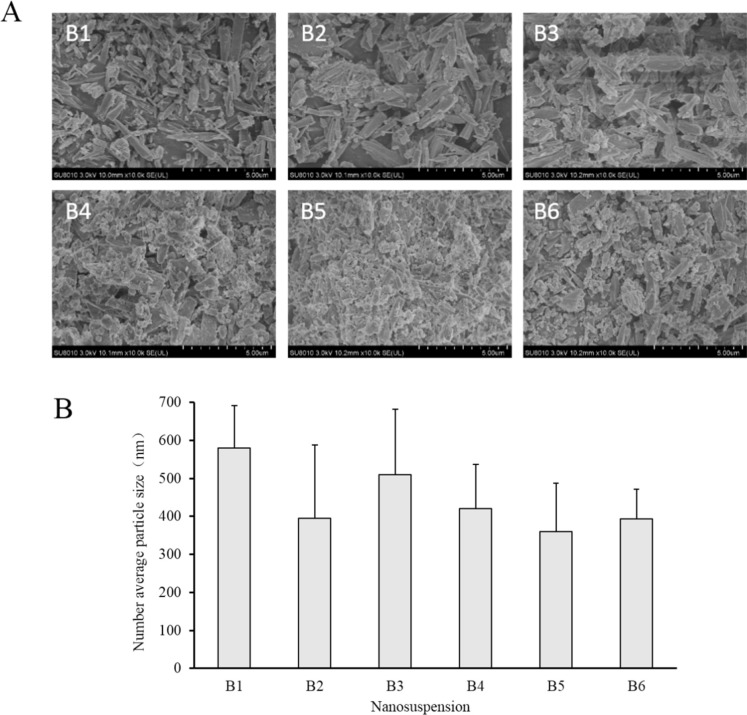
(A) SEM images of particles of sodium bicarbonate centrifuged from the corresponding nanosuspensions. (B) Number average particle size of sodium bicarbonate nanosuspensions

**Figure 2:**
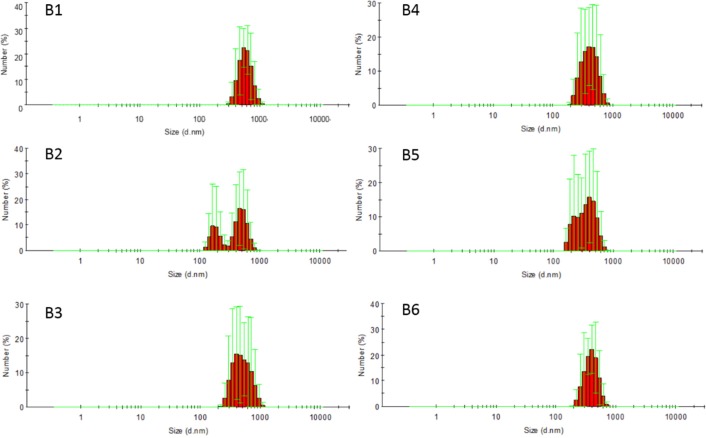
Size distribution of nanosuspensions of sodium bicarbonate (mean ± SD, n = 3)

In this study, sodium bicarbonate was firstly dissolved in a small amount of water, then sodium bicarbonate nanosuspensions (B2 to B6) were obtained by adding sodium bicarbonate aqueous solution to acetonitrile solution of ethyl cellulose with different stabilizers. B1 without any stabilizer was used as a contrast. Prescription of nanosuspensions was shown in Table 1 and the particle size, morphology and size distribution of nanosuspensions were shown in Figures 1 and 2, respectively. Number average particle sizes of nanosuspensions are all smaller than 600 nm, which is beneficial for the entrapment of sodium bicarbonate into microspheres. Particles appeared to be rod-like and non-uniform in size and various samples demonstrated no significant difference in morphology.

*Effect of stabilizer of internal oil phase on microspheres*

**Figure 3:**
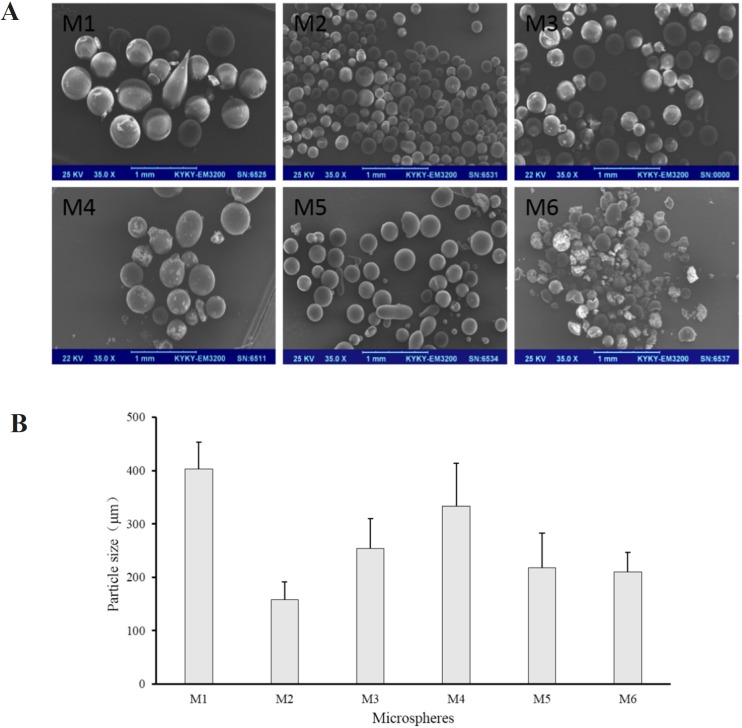
(A) SEM images and (B) number average particle size of M1 to M6

**Figure 4:**
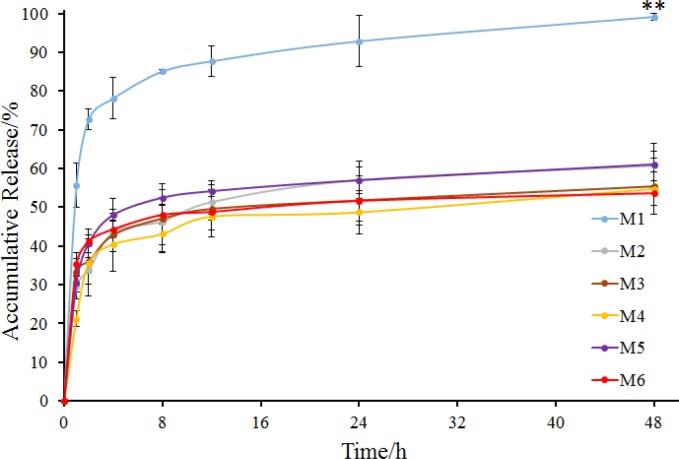
*In-vitro *release of M1 to M6 (mean ± SD, n = 3). **P < 0.01 indicated the accumulative release of M1 compared with that of other groups (M2 to M6) at 48 h

In this study, soybean oil was used as the external oil phase, and span 80 was added to soybean oil as an emulsifier because of its low HLB (4.3) and great solubility in soybean oil (28-30). The sodium bicarbonate suspensions containing Tween 20, Tween 60, Tween 80, PEG400 and Pluronic F-68 as stabilizers respectively were added to the external oil phase to form an S/O/O emulsion and then acetonitrile was volatilized to fabricate microspheres (M2 to M6). M1 without any stabilizer was used as a contrast. Prescription of microspheres was shown in Table 2 (M1 to M6). Recovery rate and drug content of microspheres were shown in Table 3 (M1 to M6). And morphology, number average particle size and *in-vitro* release of microspheres were shown in Figures 3 and 4, respectively.

M1 to M6 exhibited high recovery rates which were all beyond 70% with different drug contents (Table 3). Microspheres with Tween 80 demonstrated the highest drug content (M4, 2.68%), while microspheres without any stabilizer demonstrated the lowest drug content (M1, 1.42%). Maybe absence of stabilizer made the internal oil phase more instable, resulting in the loss of sodium bicarbonate nanoparticles from the internal oil phase. Besides, the microspheres were all spherical, but M1 had a rough surface and larger average particle size compared to other microspheres (Figure 3). The possible reason was that the addition of stabilizers into the internal oil phase could decrease the interfacial tension between the internal and external oil phase, through interaction between the hydrocarbon chains of Span 80 molecules and the solvent soybean oil via Van der Waals forces and between the multiple hydroxyl groups of Span 80 molecules and ethoxylate groups of stabilizers, in the internal oil phase via hydrogen bonds (31). Thus, in the preparation of M1, the system could be unstable and the droplets of the internal oil phase could be more uneven due to the absence of stabilizer, resulting in a larger particle size and rough surface. In the release study, M1 showed a burst release with accumulative release percentage of more than 70% in 2 h (Figure 4). M2 to M6 also had the burst release, but their release behavior showed no significant difference. It is noteworthy that the drug accumulative release of M2 to M6 at 48 h was all about 50%. Perhaps the addition of stabilizers made the microspheres denser, resulting in slower release.

**Table 1:** Prescription of nanosuspensions of sodium bicarbonate

Stabilizer								
**Nanosuspension**	**80 (g/L) NaHCO** **3** **solution (mL)**	**Acetonitrile** **(mL)**	**EC** **(mg)**	**Tween 20** **(mg)**	**Tween 60** **(mg)**	**Tween 80** **(mg)**	**PEG400** **(mg)**	**F-68** **(mg)**
B1	0.5	5	250					
B2	0.5	5	250	50				
B3	0.5	5	250		50			
B4	0.5	5	250			50		
B5	0.5	5	250				50	
B6	0.5	5	250					50

**Table 2:** Prescription of sodium bicarbonate-loaded EC microspheres

**Internal oil phase**									**External oil phase**			
	**Href :**	**H**_2 _ **O**	**EC**	**Acetonitrile**	**Tween 20**	**Tween 60**	**Tween**	**PEG400**	**F-68**	**Soybean oil**	**Span 80**	**Temperature** **(°C)**
	**(mg)**	**(mL)**	**(mg)**	**(mL)**	**(g)**	**(g)**	**(g)**	**(g)**	**(g)**	**(mL)**	**(g)**	
M1	40	0.5	250	5						45	1.5	25
M2	40	0.5	250	5	0.05					45	1.5	25
M3	40	0.5	250	5		0.05				45	1.5	25
M4	40	0.5	250	5			0.05			45	1.5	25
M5	40	0.5	250	5				0.05		45	1.5	25
M6	40	0.5	250	5					0.05	45	1.5	25
M7	80	1.0	500	10						40	1.5	25
M8	80	1.0	500	10	0.1					40	1.5	25
M9	80	1.0	500	10		0.1				40	1.5	25
M10	80	1.0	500	10			0.1			40	1.5	25
M11	40	0.5	250	5			0.05			45	1.5	30
M12	40	0.5	250	5			0.05			45	1.5	35
M13	40	0.5	250	5			0.05			45	1.5	40

**Table 3:** Recovery rate and drug content of microspheres

**Microspheres**	**Recovery rate (%)**	**Drug content (w/w, %)**
M1	89.8	1.42
M2	72.0	1.46
M3	73.6	1.51
M4	80.6	2.68
M5	87.2	2.12
M6	74.8	2.05
M7	78.1	1.76
M8	88.5	1.70
M9	74.3	1.76
M10	89.6	1.96
M11	87.0	2.93
M12	86.4	2.45
M13	88.3	2.31

**Figure 5:**
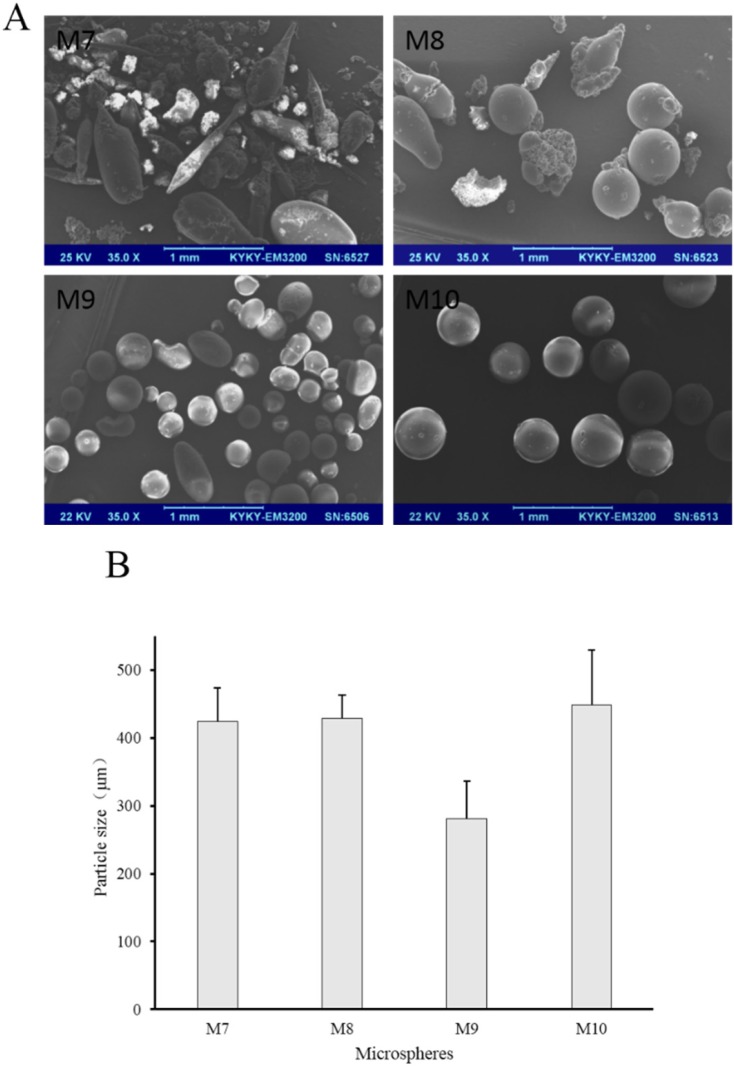
(A) SEM images and (B) number average particle size of M7 to M10

**Figure 6:**
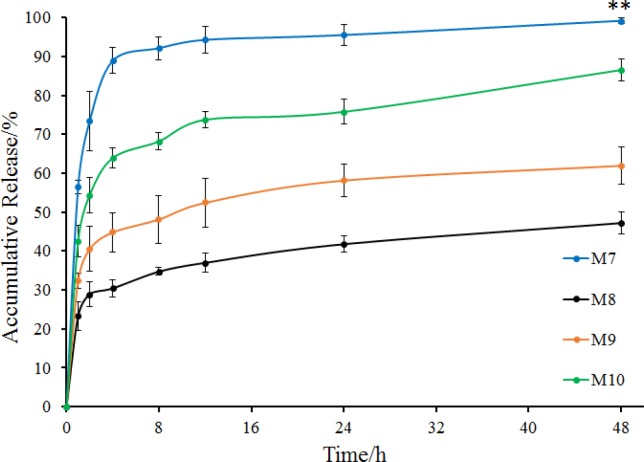
*In-vitro *release of M7 to M10 (mean ± SD, n = 3). **P < 0.01 indicated the accumulative release of M7 compared with that of other groups (M8 to M10) at 48 h

**Figure 7:**
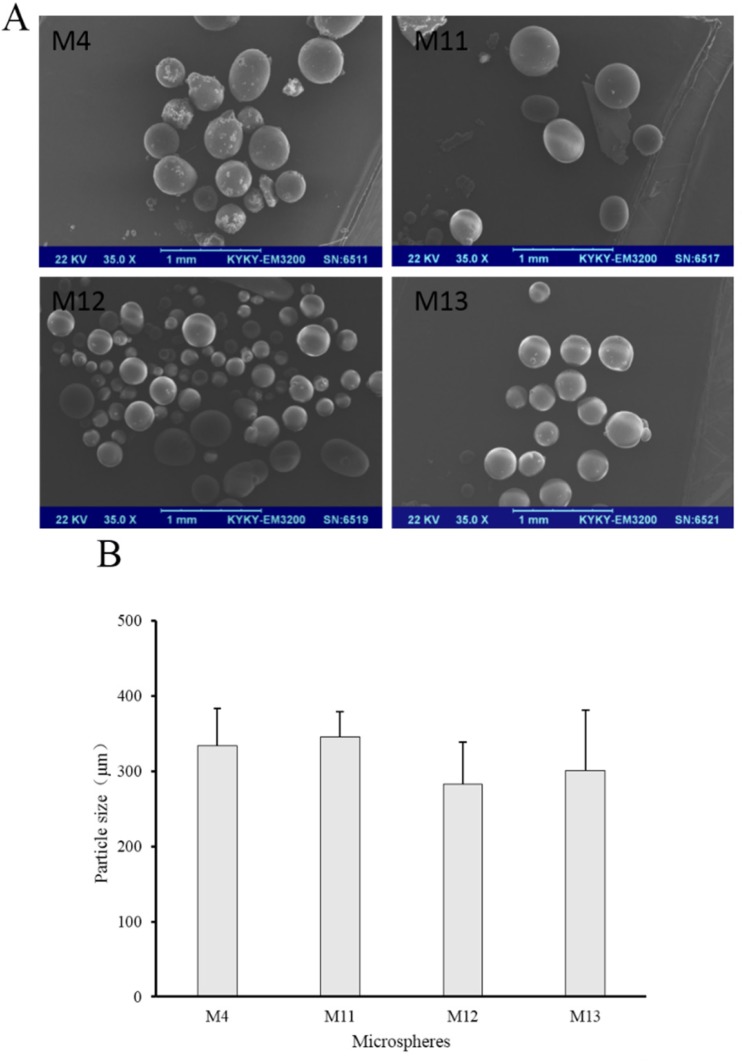
(A) SEM images and (B) number average particle size of M4, M11, M12 and M13

**Figure 8:**
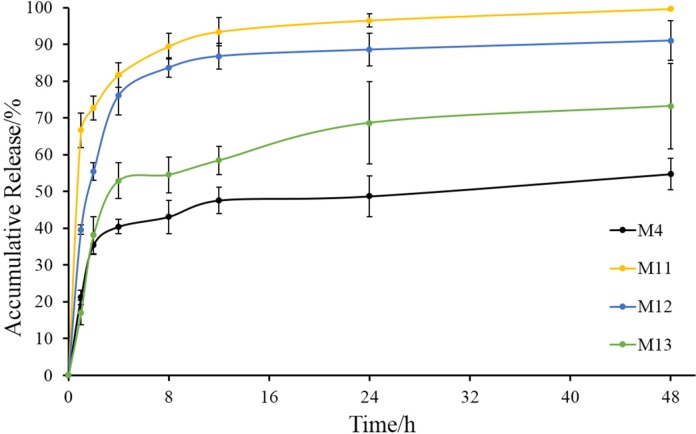
*In-vitro *release of M4, M11, M12 and M13 (mean ± SD, n = 3)

*Effect of the ratio of internal and external oil phase on microspheres*

To investigate the effect of the ratio of internal and external oil phase on microspheres, the ratio was increased from 1/9 to 1/4 with concentration of sodium bicarbonate, stabilizers and EC in acetonitrile constant. Prescription of microspheres was shown in Table 2 (M7 to M10). Recovery rate and drug content were shown in Table 3 (M7 to M10). Morphology, number average particle size and *in-vitro* release of microspheres were shown in Figures 5 and 6, respectively.

The recovery rate of M7 to M10 was not significantly different with that of M1 to M4, but the drug content changed. The drug content of M7, M8 and M9 was higher when compared with M1, M2, and M3, respectively. This may be due to the increase of the viscosity of the emulsion. But what is interesting is that the drug content of M10 was lower than that of M4. Maybe the longer stirring time due to the increased acetonitrile volume produced a relatively larger impact, resulting in more loss of sodium bicarbonate. However, the drug content of M10 was still the highest among M7 to M10, indicating that Tween 80 is advantageous as a stabilizer for drug loading. Otherwise, M7 was not spherical but rod-like, and the surface was very rough. In this case, M8, M9, and M10 still had spherical shape (Figure 5), further demonstrating the stability of the stabilizer in internal oil phase for the entire emulsion. Besides, increasing the content of internal oil phase brought larger particle size of microspheres when M7, M8, M9, and M10 compared with M1, M2, M3 and M4, respectively (Figures 3 and 5). During the preparation of the S/O/O emulsion, the stirring rate was constant, so that the shear force of the whole system did not change. But the uniformity and dispersion of the droplets could be affected when the volume of the internal oil phase increased and the mixing time was longer. These changes of the process could affect the particle size, morphology, and drug loading of the microspheres.

Release behavior of M7 to M10 was shown in Figure 6. Microspheres without stabilizer (M7) were also observed to have a burst release in which the accumulative release of 2 h was more than 70% and the accumulative release of 4 h was nearly 90%. Comparing the two groups of microspheres containing Tween 20 as a stabilizer, drug in M8 released more slowly than that in M2, and the accumulative release at 48 h was less than 50%. However, the two groups of microspheres containing Tween 60 were almost identical in their release behavior. As for the two groups of microspheres containing Tween 80, M10 exhibited a better release behavior, with a sustained release of more than 40 h with an accumulative release of 85% at 48 h (Figures 4 and 6). It seems that after increasing the ratio of internal and external oil phase, the microspheres prepared by different stabilizers produced a large difference in drug release behavior. Tween 80 showed better effect in the preparation of microspheres, with the obtained microspheres had better release behavior while maintaining high drug content.

*Effect of the emulsification temperature on microspheres*

Volatilizing acetonitrile is an important step in solvent evaporation process to fabricate microspheres. The emulsification temperature at which acetonitrile was evaporated may influence the properties of microspheres, so the emulsification temperature was investigated in our study. In this series of experiments (M4, M11, M12 and M13), Tween 80 was used as a stabilizer in the internal oil phase, because it was much more conducive to the preparation of microspheres in the previous experiment. Prescription of microspheres was shown in Table 2 (M4, M11, M12 and M13). Recovery rate and drug content were shown in Table 3 (M4, M11, M12 and M13). Morphology, number average particle size, and *in-vitro* release of microspheres were shown in Figures 7 and 8, respectively.

The drug contents of M4, M11, M12, and M13 were all beyond 2.3% and M11 had the highest drug content which was 2.93% (Table 3). Higher temperatures lead to faster evaporation of acetonitrile. It seems that 30 °C was advantageous for the drug loading. The SEM images exhibited that the increase of temperature did not cause significant changes in morphology. Microspheres of M4, M11, M12, and M13 were all spherical. 

Besides, their number average particle sizes were relatively close, which were all between 280 μm and 340 μm (Figure 7). In the release experiment, M11 showed a burst release with accumulative release of 66% in the first hour and a sustained release in the remaining time. M12 had a slower drug release than M11, especially within the initial 4 h. Drug in M13 released more slowly than M11 and M12, but the accumulative release of drugs within 48 h was 70%. As for M4, the release behavior was similar with that of M13 within the initial 2 h, but drug accumulative release of M4 at 48 h was about 50%.

## Conclusion

A novel solid-in-oil-in-oil emulsification method was successfully developed to prepare sodium bicarbonate-loaded EC microspheres and the effect of different preparation techniques was investigated. In this method, aqueous solution of sodium bicarbonate was first injected into the acetonitrile solution of ethyl cellulose to form nanosuspensions. The nanosuspensions were then added to soybean oil containing Span 80 and acetonitrile was volatilized to form microspheres. After evaluating the physical and chemical properties of the microspheres, it was found that Tween 80 as a stabilizer was beneficial to increase the drug content, and it resulted in a sustained drug release behavior when the ratio of internal and external oil phase was 1:9 or 1:4. Otherwise, different emulsification temperatures resulted in different drug release behaviors of microspheres obtained. The method in this study provided a novel idea for the encapsulation and sustained release of other thermal-instable and water-soluble drugs with low molecule weight.
